# SARS-CoV-2 crosses the blood–brain barrier accompanied with basement membrane disruption without tight junctions alteration

**DOI:** 10.1038/s41392-021-00719-9

**Published:** 2021-09-06

**Authors:** Ling Zhang, Li Zhou, Linlin Bao, Jiangning Liu, Hua Zhu, Qi Lv, Ruixue Liu, Wei Chen, Wei Tong, Qiang Wei, Yanfeng Xu, Wei Deng, Hong Gao, Jing Xue, Zhiqi Song, Pin Yu, Yunlin Han, Yu Zhang, Xiuping Sun, Xuan Yu, Chuan Qin

**Affiliations:** NHC Key Laboratory of Human Disease Comparative Medicine, Beijing Key Laboratory for Animal Models of Emerging and Remerging Infectious Diseases, Institute of Laboratory Animal Science, Chinese Academy of Medical Sciences and Comparative Medicine Center, Peking Union Medical College, Beijing, China

**Keywords:** Experimental models of disease, Blood-brain barrier

## Abstract

SARS-CoV-2 has been reported to show a capacity for invading the brains of humans and model animals. However, it remains unclear whether and how SARS-CoV-2 crosses the blood–brain barrier (BBB). Herein, SARS-CoV-2 RNA was occasionally detected in the vascular wall and perivascular space, as well as in brain microvascular endothelial cells (BMECs) in the infected K18-hACE2 transgenic mice. Moreover, the permeability of the infected vessel was increased. Furthermore, disintegrity of BBB was discovered in the infected hamsters by administration of Evans blue. Interestingly, the expression of claudin5, ZO-1, occludin and the ultrastructure of tight junctions (TJs) showed unchanged, whereas, the basement membrane was disrupted in the infected animals. Using an in vitro BBB model that comprises primary BMECs with astrocytes, SARS-CoV-2 was found to infect and cross through the BMECs. Consistent with in vivo experiments, the expression of MMP9 was increased and collagen IV was decreased while the markers for TJs were not altered in the SARS-CoV-2-infected BMECs. Besides, inflammatory responses including vasculitis, glial activation, and upregulated inflammatory factors occurred after SARS-CoV-2 infection. Overall, our results provide evidence supporting that SARS-CoV-2 can cross the BBB in a transcellular pathway accompanied with basement membrane disrupted without obvious alteration of TJs.

## Introduction

Patients infected by SARS-CoV-2 have been frequently reported to show neurological manifestations, including headache, anosmia, ageusia, impaired consciousness, seizure, stroke, and vascular events.^1,2^ Emerging evidences have revealed that SARS-CoV-2 can infect the central nervous system (CNS) in addition to the respiratory system. Huber and colleagues detected SARS-CoV-2 RNA in multiple organs, including post-mortem brain tissue of patients with COVID-19.^3^ A recent study of COVID-19 patients after an autopsy showed that SARS-CoV-2 was present in cortical neurons.^4^ In addition, the results from human brain organoids also demonstrated the neuroinvasion capability of SARS-CoV-2.^5,6^ Together, these data provide evidence for SARS-CoV-2 infection in the human CNS, but how the virus enters the brain is still unknown.

Studies have indicated that the SARS-CoV-2 virus may retrograde axonal travel from the periphery into the CNS via the olfactory sensory neurons (or other nerve tracts).^7–10^ However, there is currently no convincing evidence and it remains controversial that the olfactory nerve is not a likely route to brain infection in COVID-19.^11^ In addition to potential CNS invasion via the olfactory route, it is potential that SARS-CoV-2 may enter the brain via the hematogenous route. SARS-CoV-2 has been reported to infect choroid plexus epithelial cells in human brain organoids and the blood-cerebrospinal fluid barrier (BCSFB) might be an entryway of SARS-CoV-2 into CNS.^12^ The blood–brain barrier (BBB), which is mainly formed by endothelial cells, is another major barrier that restricts the entry of pathogens, including viruses or virus-infected cells from the systemic circulation to the CNS. Interestingly, Meinhardt et al. observed SARS-CoV-2 spike protein (S) in the endothelial cells of small CNS vessels in individuals with COVID-19 by immunostaining.^9^ Using intravenously injected radioiodinated S1, Rhea et al. indicated that the S1 protein of SARS-CoV-2 crossed the BBB in mice by adsorptive transcytosis.^13^ The SARS-CoV-2 S protein was recently demonstrated to alter BBB function in 2D static and 3D microfluidic in vitro models and to decrease BBB integrity.^14,15^ Nevertheless, there was no direct evidence that live SARS-CoV-2 could cross the BBB and it remains unresolved how the virus passes through the BBB. Here, using pathology techniques, including fluorescence in situ hybridization (FISH), transmission electron microscopy (TEM), and immunostaining, we demonstrated that SARS-CoV-2 might cross the BBB by directly BMECs infection accompanied with MMP9-mediated basement membranes disruption, without affecting tight junctions both in vivo and in vitro.

## Results

### SARS-CoV-2 infected brain vascular endothelial cells and caused brain damage in animal models

**Fig. 1:**
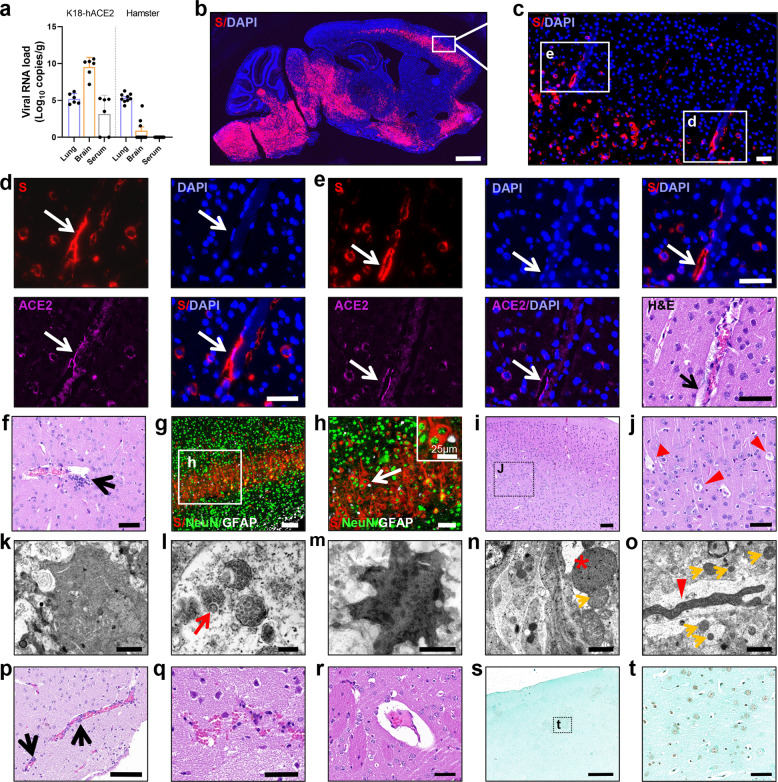
SARS-CoV-2 invades the brain and infects brain vascular endothelial cells, leading to brain damage in animal models. **a** Viral load in lung and brain tissues of SARS-CoV-2-challenged K18-hACE2 mice (*n* = 6/group) and hamsters (*n* = 9/group) was detected by qRT-PCR. **b** Distribution of SARS-CoV-2 spike protein (S) in SARS-CoV-2-challenged K18-hACE2 mice detected by fluorescence in situ hybridization (FISH). **c**–**e** FISH images showing SARS-CoV-2 co-localized with ACE2, distributed in vascular endothelial cells and in the perivascular spaces (white arrows) in the cortex of infected K18-hACE2 mice. Images of H&E-stained sequential sections subjected to FISH (**e**) showing homogeneous red-staining exudate in perivascular space (black arrow). **f** Representative H&E-stained image showing vasculitis in the brain of infected K18-hACE2 mice. Black arrow: inflammatory cell infiltration. **g**, **h** Representative images showing SARS-CoV-2-S mainly co-localized with NeuN, a marker for neurons, rather than GFAP, a marker for astrocytes in infected K18-hACE2 mice using FISH. **i**, **j** Representative images of H&E-stained neurons showing denatured and necrotic cells (red arrowhead) in the cortex layer IV of infected K18-hACE2 mice. **k** Representative ultrastructural images showing death cells undergoing pyknosis and virus-like particles (red arrow) **l** in the cortex of infected K18-hACE2 mice. **m** Pyknotic cells were also found in the cortex of infected hamsters. **n** Representative ultrastructural images showing enlarged mitochondria at high density (asterisk) and giant mitochondria **o** (red arrowhead) in the cortex of infected hamsters. Yellow arrow: normal mitochondria. **p**–**s** Representative H&E-stained images showing vascular events in the brains of infected hamsters, including perivascular inflammatory cell infiltration (**p**), focal hemorrhage (**q**), and enlarged perivascular spaces (**r**). **s**, **t** TUNEL-stained images showing apoptotic cells in the cortex of one infected hamster. **t** High magnification view of the zone delimited by the rectangle of panel **s**. Scale: **b** 1 mm; **c**–**f**, **h**, **j**, **q**, **r**, **t** 50 μm; **g**, **i**, **p** 100 μm; **k**, **m** 2 μm; **l** 200 nm; **n**, **o** 1 μm; **s** 500 μm

To confirm the neuroinvasive capacity of SARS-CoV-2 in vivo, we first examined viral load in the lung, brain, and serum after intranasal administration of SARS-CoV-2 by using K18-hACE2 transgenic mice and Syrian hamsters (Fig. 1a). Consistent with previous reports, we observed increasing viral loads in the brain tissue of SARS-CoV-2-challenged K18-hACE2 mice at 5 dpi, even higher than that in lung tissue.^16^ Viral RNA was also detected in the serum of 67% (4/6) of the challenged K18-hACE2 mice, which suggests viremia exists in these model mice. It has been suggested that brain infection observed in K18-hACE2 mice is likely linked to the aggressiveness of this model as opposed to the natural course of the viral infection. And Syrian hamsters have been reported to authentically model clinical features of severe human COVID-19 pathogenesis. Therefore, we also checked SARS-CoV-2 infected hamsters. Compared to K18-hACE2 mice, viral load was lower but it was detectable in the brains of 30% (3/9) of the challenged animals without obvious viremia at 7 dpi. These three hamsters were used for the next histological examination.

Similar to previous studies, we observed a wide distribution of SARS-CoV-2 spike protein (SARS-CoV-2-S) in the brains of K18-hACE2 mice, especially in layers III–V of the cortex, hypothalamus, midbrain, pons, and medulla co-localized with ACE2 using FISH (Fig. 1b and Supplementary Fig. 1a).^17^ Besides, we found the co-localization of SARS-CoV-2-S with blood vessels in the cortex of K18-hACE2 mice (Fig. 1c–e). As shown in Fig. 1d, the co-localization of SARS-CoV-2-S with the flattened endothelial nucleus was clearly visible. SARS-CoV-2-S was also seen in the vascular wall and perivascular space (Fig. 1e). Moreover, H&E staining of sequential sections subjected to FISH (Fig. 1e) showed a homogeneous red-staining exudate in the perivascular space of the infected vessel. These data indicate that SARS-CoV-2 may infect brain endothelial cells, leading to increased vascular permeability, which supports the probability that SARS-CoV-2 crosses the BBB. The co-localization of SARS-CoV-2-S with ACE2 in the vessels and cells suggests the role of ACE2 as the entry receptor for SARS-CoV-2 invading the brain by passing through the BBB (Fig. 1c–e). Additionally, we observed perivascular inflammatory cell infiltration in the brain of infected K18-hACE2 mice (Fig. 1f). On the whole, we found that the vast majority of viruses co-localized with NeuN (a marker for neurons), but not GFAP (a marker for astrocytes) by FISH examination (Fig. 1g, h), leading to neuronal death as shown in Fig. 1i, j, k.

To further verify the central nervous system effects of SARS-CoV-2, transmission electron microscopy (TEM) was performed. Indeed, virus-like particles appeared in the cortex of K18-hACE2 mice (Fig. 1l). In hamsters, although we did not detect clear SARS-CoV-2-S RNA by FISH, ACE2 can be slightly found in blood vessels, meninges, choroid plexus, and ependyma (Supplementary Fig. 1b). Meanwhile, pyknotic cells, abnormal mitochondrial ultrastructure, vascular injury, and cell apoptosis were detected (Fig. 1m–t). Collectively, these results favor the hypothesis that SARS-CoV-2 may have neurotrophic properties and induce brain damage by crossing the BBB in animal models.

### Activation of glial cells in SARS-CoV-2-infected animal models

**Fig. 2:**
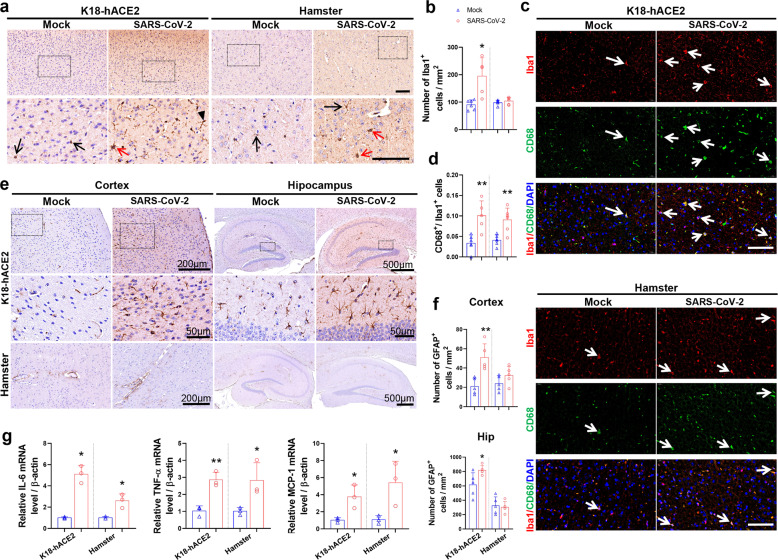
Activation of glial cells in SARS-CoV-2-infected animal models. **a** Representative images showing IHC staining for Iba1, a marker for microglia in the cortex of infected K18-hACE2 mice and hamsters. Black arrow: unactivated microglia; red arrow: M1 microglia; black arrowhead: M2 microglia. **b** Semi-quantitative analysis of microglial cells in the cortex of infected K18-hACE2 mice and hamsters. **c** Representative images showing co-localization of Iba1 and CD68 (a marker for M1 microglia) in the cortex of infected hamsters. **d** The proportion of Iba1 and CD68 double-positive microglia in the cortex of animal models. **e** Representative images showing IHC staining for GFAP, a marker for activated astrocytes in the cortex and hippocampus of infected animal models. **f** Semi-quantitative analysis of GFAP^+^ astrocytes. *N* = 5–8 slices from three animals per group. **g** Relative mRNA levels of inflammatory factors in the brains of animal models (*n* = 3/group). The *p*-values were determined by a two-tailed unpaired Students’ *t*-test. *p < 0.01,**p < 0.01, vs. mock-treated group, respectively. Scale bars: **a** 100 μm; **c** 50 μm

To further elucidate the neuropathological consequences of SARS-CoV-2 infection, we evaluated the neuroinflammatory responses, including activation of glial cells (Fig. 2a–f) and levels of inflammatory factors (Fig. 2g). The expression of Iba1, a marker for microglia, was evidence of microglial cell activation and proliferation in K18-hACE2 mice infected with SARS-CoV-2 (Fig. 2a, b). There was no significant increase in the number of microglia in infected hamsters, but microglial activation (M1 and M2) was noticeable by morphological observation. We also tested for CD68, a marker of activated microglia (M1), and found that the number of Iba1-positive cells co-staining for CD68 was increased in infected K18-hACE2 mice and hamsters (Fig. 2c, d). With respect to astrocytes, there was distinct activation in the cortex and hippocampus of infected K18-hACE2 mice compared to the mock-treated group, but little activation or proliferation in infected hamsters (Fig. 2e, f). Inflammatory factors, such as IL-6, TNF-ɑ, and MCP1, have been reported to be elevated in COVID-19 patients and models.^18^ In the present study, we also detected increased levels of IL-6, TNF-ɑ, and MCP1 in the brain of infected K18-hACE2 mice and hamsters by RT-PCR (Fig. 2g).

### SARS-CoV-2 disrupts the BBB by damaging basement membranes without affecting tight junctions

**Fig. 3:**
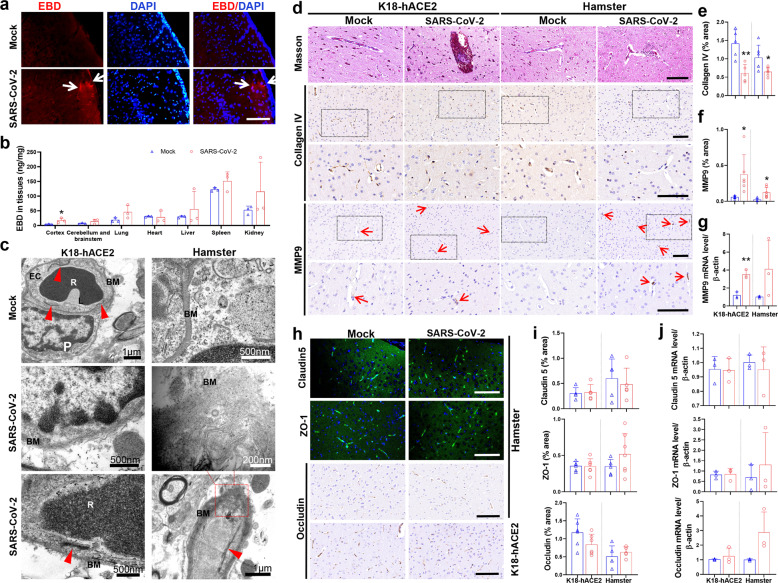
BBB disruption without tight junction impairment in SARS-CoV-2-infected animal models. **a** Representative images showing vessel leakage in the cortex of SARS-CoV-2-infected hamsters assessed by Evans blue dye (EBD), which emits red fluorescence under fluorescence microscopy. **b** Quantification of EBD in the tissues of hamsters (*n* = 3/group). **c** Ultrastructural images showing the destruction of basement membrane (BM) in the cortex after SARS-CoV-2 infection without obvious tight junction (TJ) damage. Red arrowhead: TJ. **d** Representative images showing Masson staining, immunohistochemical (IHC) staining for collagen IV and MMP9. Semi-quantitative analysis of area fraction of **e** collagen IV and **f** MMP9 staining measured by ImageJ software. **g** Relative mRNA levels of MMP9 in the brains of animal models. **h** Representative images showing immunostaining for TJ-related proteins, including claudin5, ZO-1, and occludin, and **i** histograms for their quantitative analysis. **j** Relative mRNA levels of claudin5, ZO-1, and occludin in the brains of animal models. *N* = 5–8 slices from three animals per group. The *p-*values were determined by a two-tailed unpaired Students’ *t*-test. *p < 0.01,**p < 0.01 vs. mock-treated group, respectively. Scale bar: **a**, **d**, **h** 100 μm

To assess BBB integrity, we administered Evans blue dye (EBD) by i.p. injection in both mock-treated and SARS-CoV-2-infected hamsters at 6 dpi and evaluated its leakage into the brain, which is a classic method for evaluating BBB integrity. Compared with the mock-treated hamsters, infected animals displayed visible leakage of the EBD in the cortex (Fig. 3a). Quantification of EBD in the brain also indicated that SARS-CoV-2-infected hamsters suffered worse BBB damage (Fig. 3b). Destruction of basement membranes (BMs) in the cortices of infected hamsters was found (Fig. 3c) without obvious TJ damage.

To further determine whether the BMs of brain vessels suffered damage, Masson staining was carried out. As shown in Fig. 2d, blue-stained collagen in BM was destroyed in the brains of infected K18-hACE2 mice and hamsters. The expression of collagen IV, a major component of BMs, was measured by IHC and found to be significantly decreased in the cerebral vessels of infected animals, compared to the mock group (Fig. 3e, f). Collagen IV can be degraded by matrix metalloproteases (MMPs), and MMP9-mediated collagen IV degradation has often been considered to contribute to BBB breakdown in viral encephalopathy.^19^ Here, the level of MMP9 expression was remarkably increased in the cerebral vessels compared to mock-treated animals (Fig. 3e, g). These results provide a clue that SARS-CoV-2 infection could damage BBB via MMP9-mediated BMs disruption.

It is well-known that there are two routes for pathogens to cross the BBB: the transcellular and paracellular pathways. The latter route is regulated by TJs between endothelial cells, but no apparent impairment in TJs was seen by TEM in the infected K18-hACE2 mice or hamsters (Fig. 3c). To further clarify this finding, we measured the levels of TJ-related proteins, including claudin5, ZO-1, and occludin by immunostaining (Fig. 3h, i) and saw no significant decrease in both of their expression and mRNA levels (Fig. 3j). Taken together, our data indicate that SARS-CoV-2 probably crosses the BBB via the transcellular pathway but not the paracellular pathway.

### SARS-CoV-2 can infect and replicate in BMECs and cross the BBB in vitro

**Fig. 4:**
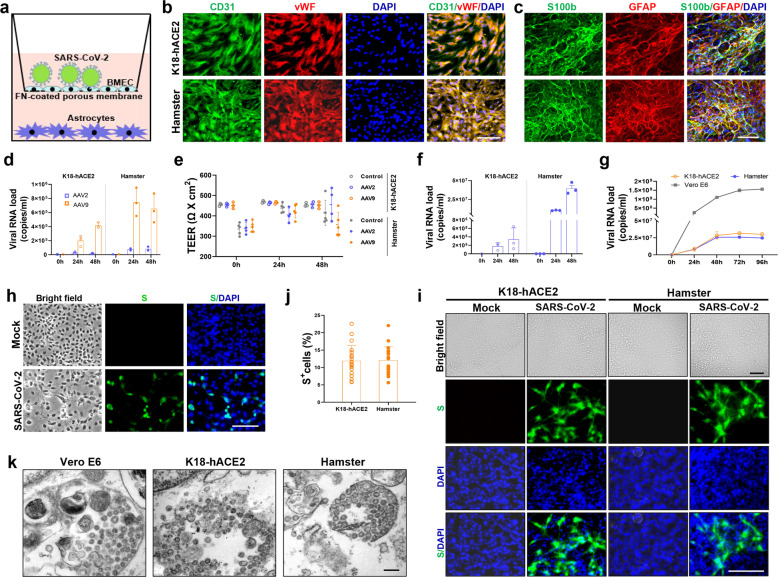
SARS-CoV-2 infected and replicated in the BMECs, and crossed the BBB in vitro*.* **a** Schematic diagram of in vitro BBB models. **b**–**e** Evaluation of in vitro BBB models, including **b** identification of primary hamster BMECs by immunofluorescent staining for CD31 and vWF, **c** immunofluorescent staining for GFAP and S100b, markers of astrocytes, the ability of AAV2 and AAV9 to cross the BBB models assessed by **d** viral RNA load in the medium from the bottom chamber and **e** transendothelial electrical resistance (TEER) in both primary BMECs from K18-hACE2 mice and hamster transwell cultures. **f** SARS-CoV-2 viral load in the medium from the bottom chamber quantified by qRT-PCR in BBB models using primary cell cultures from K18-hACE2 mice and hamsters. **g** Viral load in the medium of BMECs and Vero E6 cells quantified by qRT-PCR. **h** Representative images of SARS-CoV-2-S (S) in Vero E6 cells at 48 hpi detected by FISH. **i** Representative images of SARS-CoV-2-S (S) in BMECs at 48 hpi detected by FISH. **j** Percentage of SARS-CoV-2-S positive (S^+^) BMECs by FISH (*n* = 20 fields/group at ×200). **k** Representative ultrastructural images showing virus particles in the BMECs and Vero E6 cell. *N* = 3/group. Scale bars: **b**, **d**, **h**, **i** 100 μm; **k** 200 nm

To verify the ability of SARS-CoV-2 to cross the BBB, we established an in vitro transwell barrier BBB model using primary BMECs and astrocytes from K18-hACE2 mice or hamsters (Fig. 4a). The BMECs were identified by immunostaining for two markers-CD31 and vWF (Fig. 4b) and primary astrocytes were identified by immunostaining for two markers-GFAP and S100b (Fig. 4c). Firstly, to validate the in vitro BBB model, the adeno-associated virus vector, AAV2, which does not appreciably cross the BBB, and AAV9, which effectively crosses the BBB, were added to the upper chamber at 1 × 10^3^gc/cell. Cultures were then incubated with the virus for 24 to 48 h. In line with previous studies, the viral load in the media collected from the lower chambers revealed that AAV9 had the ability to cross our in vitro model more efficiently than AAV2 (Fig. 4d), while the TEER was not influenced by AAV2 or AAV9 (Fig. 4e). Further, to assess the ability of SARS-CoV-2 to cross the BBB in vitro, the SARS-CoV-2 virus at an MOI of 1 was added to the media in the apical transwell. The viral loads from the medium in the basal chamber at 24–48 hpi were quantified by qRT-PCR. If SARS-CoV-2 particles could pass through barrier cells, they would exist in the basal chamber. The results showed clearly that the SARS-CoV-2 viral RNA could be detected in the basal chamber in both hamsters and K18-hACE2 mice in vitro models (Fig. 4f). Take account of the lower MOI infection and more viral loads in the basal wells than AAV9, SARS-CoV-2 seems to cross the BMECs barrier much more effectively than AAV9.

To confirm whether SARS-CoV-2 can infect brain vascular endothelial cells, the BMECs were infected with SARS-CoV-2 at an MOI of 1, and allowed to grow for 24–96 hpi. As exhibited in Fig. 4g, SARS-CoV-2 infected and replicated in the BMECs and the viral RNA load in compartment supernatants peaked at 48 hpi and maintained a steady level up to 96 h. Using FISH, we detected SARS-CoV-2-S in Vero E6 and BMECs at 48 hpi (Fig. 4h–j). Furthermore, we confirmed the virus particles under TCM in Vero E6 and BMECs at 48 hpi (Fig. 4k). These results showed that SARS-CoV-2 could infect and replicate in primary BMECs from both K18-hACE2 mice and hamsters. Notably, BMECs generated a much lower titer of SARS-CoV-2 than Vero E6 cells and we did not observe any evident cytopathic effect to BMECs under the light microscope during the post-infection period (Fig. 4i).

### SARS-CoV-2 crossed the in vitro BBB without tight junction impairment

**Fig. 5:**
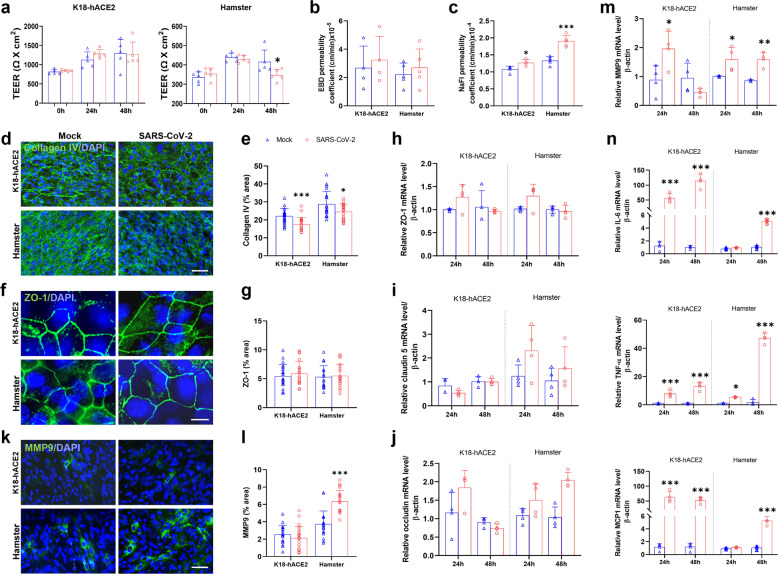
SARS-CoV-2 disrupted BBB without tight junction impairment in vitro. **a** The transendothelial electrical resistance (TEER) was recorded in primary transwell cultures of BMECs from both K18-hACE2 mice and hamsters infected at an MOI = 1 (*n* = 5/group). **b** The permeability of Evans blue dye (EBD) (*n* = 4/group) and **c** fluorescein sodium (NaFl) in the K18-hACE2 mice and hamster BBB models at 48 hpi (*n* = 5/group). **d** Representative images showing immunofluorescent staining for collagen IV in BMECs at 48 hpi. **e** Semi-quantitative analysis of area fraction of collagen IV immunofluorescent staining (*n* = 20 fields/group at ×200). **f** Representative images showing ZO-1 immunofluorescent staining in BMECs at 48 hpi and **g** its semi-quantitative analysis. **h**–**j** Relative mRNA levels of TJ-related proteins, including (**h**) ZO-1, claudin5 (**i**), and **j** occludin detected by RT-PCR (*n* = 4/group). **k** Immunofluorescence images for MMP9 in BMECs at 48 hpi. **l** Histograms for MMP9 quantitative analysis (20 fields/group were analyzed at ×400). **m** Relative mRNA level of MMP9. **n** Relative mRNA level of inflammatory factors in the medium from the upper chamber at 48 hpi (*n* = 4/group). The *p*-values were determined by two-way ANOVA (**a**, **h**–**j**, **m**, **n**) and two-tailed unpaired Students’ *t-*test (**b**, **c**, **e**, **j**, **l**). *p < 0.01, **p < 0.01, ***p < 0.001. Scale bars: **d**, **k** 50 μm; **f** 10 μm

To determine whether SARS-CoV-2 disrupted the BBB integrity in vitro, the TEER and the permeability to EBD and NaFl were measured. There was no significant TEER change in BBB models from K18-hACE2 mice. In BBB models from hamster, the TEER was decreased slightly at 48 hpi compared to the mock-treated group (Fig. 5a). And we did not observe the altered permeability to EBD in both hamster and mouse in vitro BBB models (Fig. 5b). But the permeability to NaFl was increased in both BBB models at 48 hpi (Fig. 5c).

To further assess the BBB impairment due to SARS-CoV-2 infection in vitro, we examined the expression of collagen IV. In line with the above in vivo results, collagen IV was decreased in the BBB models using BMECs from both K18-hACE2 mice and hamsters at 48 hpi (Fig. 5d, e). Meanwhile, the levels of the TJ-related proteins, ZO-1, claudin5, and occludin, were unchanged after SARS-CoV-2 infection in vitro (Fig. 5f–j). Moreover, both the mRNA and protein levels of MMP9 were increased in the BMEC BBB models at 48 hpi compared with the mock group (Fig. 5k–m). Identical to the results in vivo, the relative mRNA levels of the inflammatory factors, IL-6, TNF-ɑ, and MCP1 were much higher in the infected group than the mock control in both in vitro models of K18-hACE2 mice and hamsters (Fig. 5n). This event might occur simultaneously or after BMECs infection, being a secondary effect leading to BBB disruption.

## Discussion

**Fig. 6:**
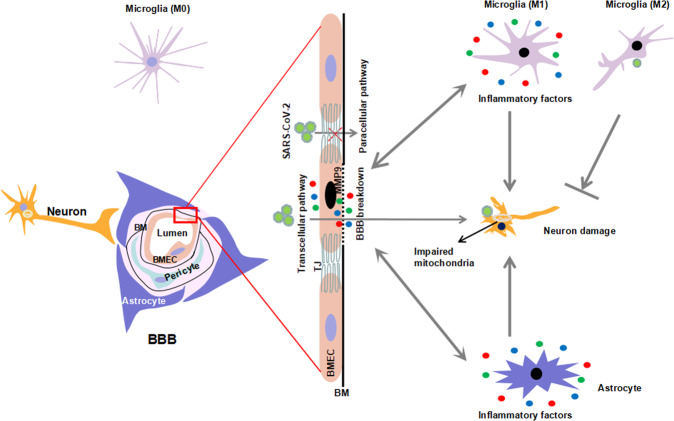
A schematic diagram depicts the possible mechanism of SARS-CoV-2 crossing the BBB. BBB is an intricate system of BMECs, pericytes, and astrocytes that can interact with neurons, microglia, and other brain components. Within BMECs, tight junctions (TJ) limit the paracellular diffusion of substances and pathogens. During infection, SARS-CoV-2 can infect the BMECs and cross the BBB to the brain via transcellular pathway by MMP9-mediated basement membrane (BM) disruption rather than a paracellular pathway. Neurons are relatively vulnerable to SARS-CoV-2 infection. Mitochondrial impairment and neuronal damage exist after infection accompanied by an inflammatory response, including activation of microglia/astrocytes and production of inflammatory factors. Further, the inflammatory factors produced by activated microglia, astrocytes and BMECs, in turn, exacerbate the damage of the BBB and neuronal injury

BBB, as an intricate system composed of BMECs, pericytes, and astrocytes, stands on the first line of defense that prevents the entry of pathogens into the brain (Fig. 6). SARS-CoV-2 was shown to have neuroinvasion ability in COVID-19 patients. Recent studies have reported vascular events, such as vasculitis, thrombosis, and endothelial disruption, as well as viremia in COVID-19, which suggests that SARS-CoV-2 may be able to pass the BBB and invade the brain.^20–23^ Here, we tested the potential for SARS-CoV-2 to cross the BBB in animal models and in cellular BBB models. We first observed the localization of SARS-CoV-2 in vascular endothelial cells and increased BBB permeability in the brains of K18-hACE2 mice infected with SARS-CoV-2. The disruption of the BBB integrity was also found in infected hamsters. The basement membranes of cerebral vessels were shown to be damaged, although the TJs were not significantly altered in either infected K18-hACE2 mice or hamsters. An increase in MMP9 expression could explain collagen degradation, resulting in the destruction of the basement membrane. Using primary BMECs and astrocytes from K18-hACE2 mice and hamsters to create in vitro BBB models, SARS-CoV-2 was observed to infect and replicate in the primary BMECs and pass through them without obvious TJ alteration. Therefore, these results implicated that SARS-CoV-2 could directly infect endothelial cells, up-regulate MMP9 to degrade basement membrane, and transcytosis through BBB to release virus particles into the parenchyma (Fig. 6).

Herein, K18-hACE2 mice and hamsters were used and the results from these two models substantially coincided except the SARS-CoV-2 and ACE2 distribution. Consistent with previous studies, SARS-CoV-2 viral RNA was detected in the brain both in K18-hACE2 mice and hamsters implicating CNS infection.^24,25^ Although we did not detect SARS-CoV-2 RNA in hamster brains by FISH, this might be due to the detection sensitivity and lower brain viral load for the absence of obvious viremia (Fig. 1a) and the relatively low level of brain ACE2 expression (Supplementary Fig. 1). However, the in vitro experiments showed SARS-CoV-2 could infect and cross BMECs both from K18-ACE2 mice and hamsters. These data suggested that SARS-CoV-2 had the ability to cross the BBB, but only proliferated in the brain under certain circumstances, such as enriched receptor expression and other necessary conditions.

It is well documented that viral infections enter into the CNS via different pathways, and multiple routes have been proposed including the BBB, cerebrospinal fluid barrier (CSF), and transneuronal transport.^26^ The BBB and BCSFB are highly complex networks to limit the passage of circulating molecules, cells, and pathogens to CNS parenchyma. And they are considered to be the major routes for virus entry into the CNS. Although it has been demonstrated that SARS-CoV-2 could infect and disrupt the BCSFB, some doubts have been raised about the involvement of the BCSFB as a major route, given that most clinical studies failed to detect significant levels of viral RNA in CSF.^12,27^ In the current study, viral RNA was primarily present in NeuN positive neurons from K18-hACE2 mice, which is consistent with a previous study from Golden et al.^25^ However, in few cases, we also detected SARS-CoV-2-S in the microvascular wall and perivascular space in the cortex of K18-hACE2 mice, indicating the possibility that the virus entry into the brain through BBB. There are two possible routes for SARS-CoV-2 to cross the BBB. One is the paracellular route by disrupting BBB integrity. It is well-known that TJs between adjacent endothelial cells form the basic structure of BBB, and play a central role in limiting virus paracellular trafficking.^28^ Here, we did not detect any significant changes in TJ proteins or loss of TJ ultrastructural integrity either in vivo or in vitro. These indicated that paracellular trafficking might not be the major way for SARS-CoV-2 across BBB (Fig. 6).

The other one for SARS-CoV-2 across the BBB is through transcytosis with or without replication in endothelial cells. Corresponding to this, SARS-CoV-2 was found to infect and cross BMECs and release virus particles to the basal chamber in in vitro BBB models, without obvious changes in TJ proteins and no significant cytopathic effect. Indeed, the basolateral release of virus particles in BMECs without significant cytopathic effect was reported in other viruses such as the Zika virus and West Nile virus.^29,30^ In line with this, recently, Rhea et al. indicated the S1 subunit of spike protein crossed the BBB by capillary bed adsorptive transcytosis and that ACE2 is involved in brain uptake.^13^

ACE2 together with TMPRSS2, cathepsins, and other SARS-CoV-2 entry factors have been demonstrated in endothelial cells, although levels of ACE2 and TMPRSS2 are lower than in nasal epithelium and pulmonary alveolar type 2 cells.^31^ However, whether the virus directly infects endothelial cells remains a matter of controversy. Varga et al. showed evidence of direct viral infection of endothelial cells and diffuse endothelial inflammation in patients with COVID-19,^32^. Similarly, Bhatnagar et al. also demonstrated direct infection of vascular endothelium by SARS-CoV-2 by localizing viral RNA in endothelial cells and the tunica media of the vessels in multiple tissues, including lungs, brain stem, cerebellar leptomeninges, heart, liver, kidney, and pancreas.^33^ Furthermore, Paniz-Mondolfi et al. have revealed the presence of SARS-CoV-2 in brain capillary endothelial cells in a COVID-19 patient.^34^ In vitro experiments also proved that SARS-CoV-2 could directly infect engineered human capillary organoids, and the infection could be inhibited by soluble human recombinant ACE2.^35^ Recently, Jiao et al. showed SARS-CoV-2 could infect neuro-derived SK-N-SH, glial-derived U251, and brain microvascular endothelial cells in vitro without efficient replication.^10^ In contrast, Schaefer et al. found no evidence of the virus in post-mortem pulmonary endothelial cells of COVID-19 patients with diffuse alveolar damage by immunohistochemical staining.^36^ And multiple studies challenge the identification of “virus-like particles” in endothelial cells under EM as coronavirus particles.^37–39^ In a recent study, it was found that primary human endothelial cells derived from lung, heart, kidney, brain, and umbilical veins were resistant to infection with SARS-CoV-2.^40,41^ These disparate findings could be due to the organ-specific heterogeneity of the endothelium, and endothelial cells show tissue- and vascular bed-specific profiles that may regulate their susceptibility and permissibility to SARS-CoV-2 infection.^12,42,43^ Besides tissue- and vascular bed-specific profiles, these inconsistent results in viral susceptibility of endothelial cells may be the result of differences in virus strain, viral inocula loads, cell culture conditions, number of cell passages, etc. And it is possible that primary endothelial cells from newborn pups may be more susceptible to infection. COVID-19-related chilblains are being seen with an exceedingly high frequency in children and young adults and the presence of SARS-CoV-2 in the endothelium of dermal vessels has been shown in skin biopsies of children, although they had no or mild systemic symptoms and SARS-CoV-2 PCR from nasopharyngeal and oropharyngeal swab was negative.^44^ The disparity could also be due to genetic differences between rodents and humans. For example, the expression of ACE2 was reported to be relatively low in endothelial cells from the human brain, whereas ACE2 expression was high in mouse brain endothelial cells.^45^ Besides, we did not found that SARS-CoV-2 infect astrocytes in the current study. This is supported by the studies of Ramani et al. who showed that SARS-CoV-2 preferably targeted neurons of brain organoids.^5^ Likewise, Jacob et al. demonstrated the ability of SARS-CoV-2 to infect human cortical neurons and astrocytes except in a few cases of only sparse infection.^46^ Whereas, Crunfli et al. found that SARS-CoV-2 infected and replicated in astrocytes and neurons, altered astrocyte metabolism and neuronal viability, and caused impairment of brain function in COVID-19 patients.^47^

However, endothelial cell infection with SARS-CoV-2 in the brain is not common. We only detected SARS-CoV-2 S RNA in BMECs from two infected K18-hACE2 mice. Even in vitro, the proportion of virus-positive endothelial cells is only about 10% (Fig. 4j), and BMECs showed much lower viral replication without cytopathic effects when compared to Vero E6 cells, which is coincident with only a scarce number of endothelial cells co-express ACE2 and TMPRSS2 mRNA by single-cell assay and the relative rarity of neuroinvasion and cerebrovascular events in COVID-19 patients.^48^ It is important to notice, there are very few studies confirming the infection of BMECs upon human natural infection indicating that SARS-CoV-2 could not be high-efficiently replicated in BMECs. Yet, it is still possible that SARS-CoV-2 infects and transcytosis through BMECs with low replication or even lack of replication just like the Zika virus and West Nile virus. These viruses across intact endothelium probably rely on properties that allow attachment and transcytosis across the intact endothelial barrier, rather than productive infection, seeing that the limited evidence that Zika virus and West Nile virus replicate in endothelial cells during natural infection.^49^

Although no changes were found in TJs, BBB permeability was changed both in vivo and in vitro BBB models. Especially, present results revealed destruction of the endothelial basement membrane with collagen IV degradation, which might be related to the up-regulation of MMP9 expression. In line with these findings, Buzhdygan et al. showed that the SARS-CoV-2 spike protein could have a direct impact by altering the barrier function in 2D static and 3D microfluidic in vitro models and by increasing matrix metalloproteinase gene expression.^14^ MMP9, a kind of zinc-ion-dependent endopeptidase, plays important role in remodeling the extracellular matrix. Clinically, it has been reported that circulating MMP9 was distinct and early up-regulated in COVID-19 patients with respiratory failure.^50^ And Kawasaki-like disease has already been linked to coronavirus infections in children while Kawasaki disease is strongly related to a viral-induced epigenetic overexpression of MMP9.^51^ It is worth noting that MMP9 may play a key role in mediating viral invasion. For West Nile virus, MMP9 was shown to partly localize to the blood vessels and its expression was up-regulated in the murine brain. Moreover, the brain viral loads, BBB permeability, inflammatory cytokines, and leukocyte infiltrates were all significantly reduced in the MMP9 knockout mice.^52^ Similarly, Hui et al. showed MMP9 facilitated Zika virus invasion of the testis by modulating the integrity of the blood–testis barrier.^53^ Additionally, it has been reported that MMP9 level increases in ACE2-knockout mice model and ACE inhibitors target MMP9 along with ACE2, indicating the interaction between ACE2 and MMP9.^54,55^ Hence, the potential MMP9-mediated effects of SARS-CoV-2 infection on CNS tissues deserve further study and that strategies to interrupt this process may influence the course of SARS-CoV-2 induced neurological manifestations.

Besides, we showed that SARS-CoV-2 infection led to an inflammatory response both in vivo and in vitro. The levels of pro-inflammatory factors, IL-6, TNF-ɑ, and MCP1 were elevated in BMECs and this may contribute to the increase in BMEC permeability. Studies have indicated that a hyperinflammatory syndrome induced by SARS-CoV-2 in COVID-19 can result in immunothrombosis, microcirculatory disturbances, multi-organ injury, and mortality. COVID-19-associated vascular inflammation, particularly endotheliitis, may explain the systemic impaired microcirculatory function in different organs in COVID-19 patients.^56^ After SARS-CoV-2 infection, endothelial cells become activated, show elevated expression of pro-inflammatory cytokines, and recruitment of inflammatory cells, which may aggravate the breach in the endothelial barrier function and induce neuroinflammation and neurological symptoms. Accordingly, we observed perivascular inflammatory cell infiltration and activation of glial cells in the brain of infected animals. And it is also possible that pro-inflammatory cytokines recruitment peripheral-infected leukocytes transmigrate between the endothelial cells and release virus within the CNS through a mechanism called “Trojan horse”.^57^ Noteworthily, we also observed M2 microglia in SARS-CoV-2 infected hamster, which may be the self-protective reaction to facilitate the virus elimination (Fig. 6).

In conclusion, our data indicated that SARS-CoV-2 could infect brain vascular endothelial cells and cross the BBB via transcellular pathway by MMP9-mediated BM with intact TJs, consequently leading to neuronal damage, which further promoting the understanding of the mechanisms underlying CNS invasion by SARS-CoV-2 and neurologic manifestations in COVID-19 patients.

## Material and methods

### Biosafety and ethics statement

All experiments with live SARS-CoV-2 were conducted in a biosafety level 3 (ABSL3) facility at the Institute of Laboratory Animal Science (ILAS), Chinese Academy of Medical Sciences, Beijing, China. The animal studies were reviewed and approved by the Institutional Animal Care and Use Committee of ILAS (QC20010).

### Virus

SARS-CoV-2 (NMDC10013001) isolates were propagated in Vero E6 cells in DMEM (Invitrogen) containing 10% fetal bovine serum (FBS), 100 IU/ml penicillin, and 100 µg/ml streptomycin, and incubated at 37 °C as previously described.^58^ AAV2 and AAV9 were obtained from Vigene Biosciences, Shandong, China. Both serotypes of AAV(AAV2-CMV-GFP and AAV9-CMV-GFP) encode the eGFP protein under the cytomegalovirus promoter.

### Animal experiments

For the animal experiments, specific pathogen-free, male K18-hACE2 transgenic mice (4–6 weeks) from GemPharmatech Co., Ltd. and Syrian hamsters (6–8 weeks) were obtained from Beijing Vital River Laboratory Animal Technology Co., Ltd. After intraperitoneal (i.p.) anesthesia with avertin (350 mg/kg), the K18-hACE2 mice were inoculated intranasally with SARS-CoV-2 stock virus at a dosage of 10^2^ TCID_50_. Hamsters were challenged with 10^5^ TCID_50_ after i.p. anesthesia with zoletil (50 mg/kg). K18-hACE2 mice and hamsters intranasally inoculated with equal volumes of PBS were used as mock-treated controls. The mice were euthanized at 5 days post-infection (dpi) and hamsters at 7 dpi, and serum, brain, and lung tissues were obtained to screen for virus replication and histopathological changes.

### Fluorescence in situ hybridization (FISH)

FISH was performed on paraffin-embedded tissue sections (5 μm) and neutral formalin-fixed cells using the RNAscope multiplex fluorescent assay v2 (#323100, ACD, USA) according to the manufacturer’s instructions. Tissue sections were deparaffinized with xylene and 100% ethanol, incubated with hydrogen peroxide (#322330, ACD, USA) for 10 min, followed by target retrieval in boiling water for 15 min, and incubation with Protease Plus for 15 min at 40 °C. Slides were hybridized with target-specific probes (Table 1) at 40 °C for 2 h, and signals were amplified according to the manufacturer’s instructions. TSA plus® fluorescein, TSA plus® cyanine 3, and TSA plus® cyanine 5 (PerkinElmer) were used at 1:1000 dilution to visualize the signals. The FISH procedures for cells were the same as above except for the ethanol pretreatment and protease III incubation. A Leica fluorescence microscope (Dmi8) was used to image and digitize the stained cells. Tissue sections were scanned using a Panoramic III scanner (3D Histech, Hungary) and digital images were obtained.

**Table 1:** Probes used for in situ hybridization

Probes	Source
RNAscope probe-Mm-NeuN-C1	#313311, ACD
RNAscope® Probe-Hs-ACE2-C2	#848151, ACD
RNAscope® Probe-V-nCoV2019-S-C3	#848561, ACD
RNAscope Probe-Mm-Gfap-C2	#313211, ACD

### Transmission electron microscopy

Ultrastructural analysis was performed as previously described.^59^ Briefly, the frontal cortex was carefully dissected out and fixed in 2.5% glutaraldehyde at 4 °C for 2 h. The samples were treated with 1% osmium tetroxide in 0.1 M cacodylate buffer for an additional 1 h, followed by 1% uranyl acetate and dehydrated in ethanol. Samples were embedded in epoxy resin, sectioned (90 nm), and placed on carbon-coated copper grids. After uranyl acetate and lead citrate staining, the specimens were visualized by transmission electron microscopy (JEOL JEM-1400).

### Histological examination

Brain tissue from mice and hamsters was fixed in 10% neutral buffered formalin, embedded in paraffin, and sectioned (4–5 μm thickness). After dewaxing, the cortical sections were washed in dH_2_O for H&E staining, modified Masson staining (#G1346, Solarbio, China), and TUNEL staining (ab206386, Abcam) according to the manufacturer’s instructions. For immunohistochemical (IHC) staining, sections were treated with an antigen-retrieval kit (Boster, AR0022) for 1 min at 37 °C and quenched for endogenous peroxidases in 3% H_2_O_2_ in methanol for 10 min After blocking in 1% normal goat serum, the sections were incubated with the specific primary antibody (Table 2) overnight at 4 °C, washed, and the immunoreaction was visualized by incubating with the secondary antibody (HRP-conjugated anti-rabbit/mouse IgG) and DAB (ZSGB-BIO, Beijing, China). The nuclei were stained with hematoxylin.

**Table 2:** The antibodies used in the present study

Antibody	Source	Host	Dilution
Collagen IV	ab236640, Abcam	Rabbit	1:500
MMP9	ab76003, Abcam	Rabbit	1:500
Claudin5	352500, Invitrogen	Mouse	1:500
ZO-1	339100, Invitrogen	Mouse	1:500
Occludin	ab216327, Abcam	Rabbit	1:500
Iba1	ab178846, Abcam	Rabbit	1:500
CD68	MA5-13324, Invitrogen	Mouse	1:200
GFAP	ab7260, Abcam	Rabbit	1:500
CD31	ab24590, Abcam	Rabbit	1:500
vWF	ab6994, Abcam	Mouse	1:500
S100b	ab218515, Abcam	Mouse	1:500
Goat anti-rabbit lgG-conjugated TRITC	ZF-0317, Beijing ZSGB Biotechnology	Goat	1:200
Goat anti-mouse lgG-conjugated TRITC	ZF-0313, Beijing ZSGB, Biotechnology	Goat	1:200
Goat anti-rabbit lgG-conjugated FITC	ZF-0311, Beijing ZSGB Biotechnology	Goat	1:200
Goat anti-mouse lgG-conjugated FITC	ZF-0312, Beijing ZSGB Biotechnology	Goat	1:200
Goat anti-rabbit lgG-conjugated FITC	ZF-0311, Beijing ZSGB Biotechnology	Goat	1:200
Goat anti-rabbit lgG secondary antibody-conjugated HRP	PV9001, Beijing ZSGB Biotechnology	Goat	1:200
Goat anti-mouse lgG secondary antibody	PV9002, Beijing ZSGB Biotechnology	Goat	1:200

For immunofluorescent staining, the sections were incubated with appropriate secondary antibodies conjugated with FITC/TRITC (Beijing ZSGB Biotechnology, 1:300). The nuclei were stained with DAPI. The sections were observed via light/fluorescence microscopy, scanned using a Panoramic III scanner (3D Histech, Hungary), and the digital images were thus obtained, analyzed with ImageJ software (v1.51j8, NIH, USA).

### In vivo assessment of BBB integrity

Changes in BBB permeability in vivo were assessed using Evans blue dye (EBD) as a marker of albumin extravasation as previously described.^60^ Briefly, hamsters were infected for six days and intravenous injections of 2% EBD solution in saline (4 ml/kg) were administered after i.p. anesthesia with zoletil (50 mg/kg). EBD was allowed to circulate for 45 min Animals were then perfused transcardially with saline until fluid from the right atrium became colorless. Brains and other major organs were removed, weighed, and the dye was extracted with formamide at 56 °C for 48 h. Dye concentration was quantified spectrophotometrically at 620 nm and normalized to the weight of the tissue. EBD fluorescence was excited using a 633 nm laser.^61^To further compare EBD extracted from the parenchyma of the brain cortex, we imaged sections by fluorescence microscopy. Brains were fixed with 4% paraformaldehyde and dehydrated for cytoprotection in 30% (wt/vol) sucrose. After the specimens were frozen, 20 μm serial cryostat sections were made for fluorescence imaging.

### Viral load quantification

Tissues homogenates (1g/ml) were prepared using an electric homogenizer for 2.5 min in DMEM. The homogenates were centrifuged at 1000 × *g* for 10 min at 4 °C. The supernatants were collected and stored at −80 °C for measuring viral load as previously described.^58^ Total RNA was extracted from tissue homogenates and reverse transcribed into cDNA. Quantitative real-time PCR (qRT-PCR) was performed using the PowerUp SYBR Green Master Mix Kit (Applied Biosystems, USA). Duplicate samples were amplified using the following cycling protocol: 50 °C for 2 min, 95 °C for 2 min, followed by 40 cycles at 95 °C for 15 s and 60 °C for 30 s. The primer sequences used for RT-PCR were targeted against the envelope (E) gene of SARS-CoV-2: forward, 5′-TCGTTTCGGAAGAGACAGGT-3′; reverse, 5′-GCGCAGTAAGGATGGCTAGT-3′. The standard curve was constructed for quantification by plotting the plasmid copy number against the corresponding threshold cycle values.

For in vitro cell experiments, cell supernatants and cells were harvested in TRIzol LS reagent or TRIzol reagent (Invitrogen) and RNA was purified following phase separation by chloroform as recommended by the manufacturer. RNA in the aqueous phase was collected and further purified using PureLink RNA Mini Kits (Invitrogen) according to the manufacturer’s protocol. Viral RNA was quantified by qRT-PCR on a StepOnePlus Real-Time PCR System (Applied Biosystems) using TaqMan Fast Virus 1-Step Master Mix chemistry (Applied Biosystems).^62^ SARS-CoV-2 ORF1ab gene RNA was amplified using forward (5′-CCCTGTGGGTTTTACACTTAA) and reverse (5′-ACGATTGTGCATCAGCTGA) primers and probe (5′-FAM-CCGTCTGCGGTATGTGGAAAG GTTATGG-BHQ1) designed by the Chinese Centers for Disease Control and Prevention. RNA copy numbers were interpolated from a standard curve produced with serial 10-fold dilutions of ORF1ab gene RNA with a known copy number.

### RNA extraction from brain and qRT-PCR

Total RNA was extracted from the whole brain or cells using TRIzol reagent (Invitrogen) following the manufacturer’s instructions. Total RNA was denatured at 65 °C for 5 min, and subjected to reverse transcription using an oligo(dT) primer and SuperScript™ III reverse transcriptase (Invitrogen™) at 50 °C for 1 h. Quantitative RT-PCR was performed on a StepOnePlus real-time PCR system (Applied Biosystems) with SYBR Green Master Mix (ThermoFisher Scientific). The amplification conditions were 95 °C for 10 min, followed by 40 repeats of 95 °C for 15 s and 60 °C for 1 min The sequences of oligonucleotides used in the study are listed in Table 3.

**Table 3:** The sequence of oligonucleotides used in RT-PCR

Name	Forward	Reverse
K18-hACE2		
Claudin5	5′-GCAAGGTGTATGAATCTGTGCT-3′	5′-GTCAAGGTAACAAAGAGTGCCA-3′
ZO-1	5′-GATGTTTATGCGGACGGTGG-3′	5′-AAATCCAAACCCAGGAGCCC-3′
Occludin	5′-GGCAAGCGATCATACCCAGA-3′	5′-TTCCTGCTTTCCCCTTCGTG-3′
MMP9	5′-CTGGACAGCCAGACACTAAAG-3′	5′-CTCGCGGCAAGTCTTCAGAG-3′
β-actin	5′-CCAGCCTTCCTTCTTGGGTAT-3′	5′-CATAGAGGTCTTTACGGATGTCAAC-3′
IL-6	5′-TTCAGAGCACCATCAAAA CCC-3′	5′-GCCACTCCTTTTGTGACTCC-3′
TNF-α	5′-CTGAACTTCGGGGTGATCGG-3′	5′-TACAGCCCGTCTGCTGGTAT-3′
MCP1	5′-AACGCTT CTGTGCCTACTGC-3′	5′-TCTTGTAGCTCTCCAGCCTCT-3′
Hamster		
Claudin5	5′-GGGCGAGCATTCGGTCTTTA-3′	5′-AGAATCAAGCCCACCCAACC-3′
ZO-1	5′-TAAACCTCCAAGTGCTTCCCT-3′	5′-CTTCAGGTGGCTTGACTTGAG-3′
Occludin	5′-ATGGGAGTCAACCCAACTGC-3′	5′-ATGGGAGTCAACCCAACTGC-3′
MMP9	5′-CGTGTGTGGAG GTTCGACTT-3′	5′-TCGTCTCGGAAACTCACACG-3′
β-actin	5′-AGAAGCTGTGCTATGTTGCCC-3′	5′-GCCACAGGA TTCCATACCCAG-3′
IL-6	5′-TAGTCCTTCCTACCCCAATTTCC-3′	5′-TTGGTCCTTAGCCACTCCTTC-3′
TNF-α	5′-CTGAGTTCTGCAAAGGGAGAG-3′	5′-CCTCAGGGAAGAATCTGGAAAG-3′
MCP1	5′-TTAAAAACCTGGATCGGAACCAA-3′	5′-GCATTAGCTTCAGATTTACGGGT-3′

### Cell culture

Primary cultures of BMECs were isolated as previously described with some modifications.^63^ K18-hACE2 mice or hamster pups (P7, mixed gender) were selected for cell isolation. Animals were euthanized, brains were removed and the cortex was cut into 1 mm^3^ blocks in DMEM. The cortex was digested in 10 ml of 0.1% type II collagenase containing 30 U/ml DNase I (Roche, Basel, Switzerland) for 90 min and the disrupted tissue was centrifuged at 300 × *g* at room temperature (RT) for 10 min Supernatants were discarded, and the tissue was resuspended in 20% bovine serum albumin and centrifuged at 1000 × *g* at 4 °C for 20 min After removal of nerve tissue and blood vessels in the upper layer, the isolated tissue was digested with 2 ml of 0.1% collagenase/dispase (Roche, Basel, Switzerland) containing 20 U/ml DNase I (Roche, Basel, Switzerland) at RT for 60 min The remaining pellets were washed once and centrifuged at 300 × *g* for 5 min The pellets were resuspended in 2 ml of DMEM plus 10% FBS, carefully layered on top of 9 ml of 50% Percoll solution and centrifuged at 1000 × *g* for 10 min at 4 °C. Bands containing microvessel fragments of the desired density were isolated and washed twice with DMEM plus 10% FBS and centrifuged at 300 × *g* for 5 min The cells were seeded into a fibronectin-coated culture flask and cultured in endothelial cell medium (Cat. #1001, ScienCell Research Laboratories, Inc.). The purity of BMECs was assessed by immunofluorescence using anti-CD31 and anti-vWF antibodies (Table 2).

Primary cultures of cortical astrocytes were prepared from K18-hACE2 mice or hamsters (P7, mixed gender) as previously described with some modifications.^64^ Briefly, cerebral hemispheres were obtained under sterile conditions, dissected free of meninges, and dissociated with 0.125% trypsin-EDTA for 20 min at 37 °C. The cell suspension was filtered through a nylon mesh with a pore size of 70 μm. Cells were seeded into a poly-l-lysine (PLL)-coated cell culture flasks in DMEM containing 10% FBS and 1% antibiotics. After 15 min, the differential attachment method was used to remove fibroblasts. The unattached cells were transferred to a new PLL-coated cell culture flask in DMEM with 10% FBS. Cultures were grown in a humidified atmosphere of 5% CO_2_/95% air at 37 °C. The purity of the astrocyte culture was assessed by immunofluorescence using anti-GFAP and anti-S100b antibodies (Table 2).

For direct infection, BMECs or Vero E6 cells were incubated with SARS-CoV-2 at an MOI of 1 for 1 h at 37 °C. The viral inoculum was removed and replaced with a fresh medium. The mock-treated controls were incubated with an equal volume of DMEM. Cells were incubated 24–48 h, or up to 96 h, according to the particular experiment. Cells were harvested by centrifugation and total RNA was extracted and reverse transcribed for qRT-PCR analysis.

### In vitro BBB model

Millicell hanging cell culture inserts with a pore size of 0.4 µm (Corning, New York, USA) were placed into 24-well plates. BMECs (1 × 10^5^/ml) were seeded on top of the transwell and incubated at 37 °C. Transepithelial electrical resistance (TEER) was monitored, and once the TEER value was constant, the chambers with BMECs were carefully placed into new 24-well plates with primary cortical astrocytes. A volume of 1.3 ml DMEM plus 10% FBS was added to the bottom (basal) wells of the companion plate, and incubated for 24 h at 37 °C in 5% CO_2_.

To validate the effects of SARS-CoV-2 in the in vitro BBB model, AAV2 (a vector that does not appreciably cross the BBB) and AAV9 (a vector that has been reported to cross the BBB effectively) were applied to the upper chamber at 1 × 10^3^ gc/cell. Cultures were then incubated with the virus at 37 °C for 24 and 48 h. At the indicated times, TEER was recorded and media was collected from the lower chambers to quantify the number of AAV genomes crossing the insert by qRT-PCR.^65^ To assess the ability of SARS-CoV-2 to cross the BBB in vitro, 300 μl of SARS-CoV-2 virus was added to the medium in the apical transwell insert at an MOI of 1. The mock-infected controls received the equivalent volume of medium without the SARS-CoV-2 virus. At 24 and 48 h post-infection, 200 μl samples of medium from the bottom wells were removed, and infectious viral load was determined by qRT-PCR.

### BBB in vitro permeability assays

TEER is a measure of barrier integrity and was determined using the Millicell ERS-2 volt-ohm meter (Millipore, Boston, MA, USA). TEER was calculated by subtracting the resistance of blank inserts from that of the inserts with cells and multiplying the subtracted values by the area of the insert. Barrier function was further analyzed by measuring the permeability of the cell monolayer to EBD-labeled albumin (67 kDa) (Sigma-Aldrich, St. Louis, MO) and fluorescein sodium salt (NaFl, 376 Da).^66^ Hank’s balanced salt solution (HBSS) was added to the abluminal side, while the luminal side was loaded with HBSS containing 170 μg/ml EBD, 20 μg/ml NaFl, and 10 mg/ml bovine serum albumin. The cells were incubated at 37 °C for 60 min, and the levels of NaFl and EBD in the abluminal side were measured using a fluorometer with excitation/emission wavelengths of 485/530 nm for NaFl and excitation/emission wavelengths of 540/680 nm for EBD. EBD and NaFl concentrations were determined using a standard curve. The permeability coefficient (*P*) for EBD and NaFl was calculated according to the equation: *P*(cm/s) = *V*_B_/(*S* × *C*_A_) × (Δ*C*_B_/Δ*t*), where *V*_B_ is the volume in the basolateral compartment, *S* is the surface area of the filter membrane, *C*_A_ is the initial concentration in the apical compartment, and Δ*C*_B_/Δ*t* is the change of concentration over time in the basolateral compartment.^67^

### Statistical analysis

All data were analyzed with GraphPad Prism 8.0 software. Statistically significant differences were determined using unpaired Student’s *t*-tests, or ANOVA tests according to test requirements. A two-sided *p*-value <0.05 was considered statistically significant. *p < 0.05, **p < 0.01, ***p < 0.001.

## Supplementary information

Supplementary Material

Supplemental Figure 1

## Data Availability

All raw data are available from the corresponding author on reasonable request.
