# Supplementary material for: SARS-CoV-2 crosses the blood–brain barrier accompanied with basement membrane disruption without tight junctions alteration
Source: Signal Transduct Target Ther. 2021 Sep 6;6:337. doi: 10.1038/s41392-021-00719-9 (PMC8419672; doi:10.1038/s41392-021-00719-9)
Supplement: Supplementary file 1 — Supplementary Material [file 41392_2021_719_MOESM1_ESM.doc]

Supplementary Materials for

**SARS-CoV-2 crosses the blood-brain barrier accompanied with basement membrane disruption without tight junctions alteration**

Zhang Ling^＃^, Zhou Li^＃^, Bao Linlin, Liu Jiangning, Zhu Hua, Lv Qi, Liu Ruixue, Chen Wei, Tong Wei, Wei Qiang, Xu Yanfeng, Deng Wei, Gao Hong, Xue Jing, Song Zhiqi, Yu Pin, Han Yunlin, Zhang Yu, Sun Xiuping, Yu Xuan, Qin Chuan^＊^

Correspondence to: [qinchuan@pumc.edu.cn](mailto:qinchuan@pumc.edu.cn)

**This PDF file includes:**

Fig. S1

**Figure. S1**. The distribution of ACE2 in brain tissues after SARS-CoV-2 infection in vivo. (a-c) Representative images showing SARS-CoV-2-S (S) widely co-distributed with ACE2, especially in layers III-V of the cortex (b), hypothalamus, midbrain, pons and medulla (c). ACE2 mainly co-localized with NeuN, a marker for neurons, in infected K18-hACE2 mice using FISH. (d) Representative images showing ACE2 mainly distributed in meninges, blood vessels, choroid plexus and ependyma in infected hamsters with no obvious SARS-CoV-2-S detection by FISH.

**
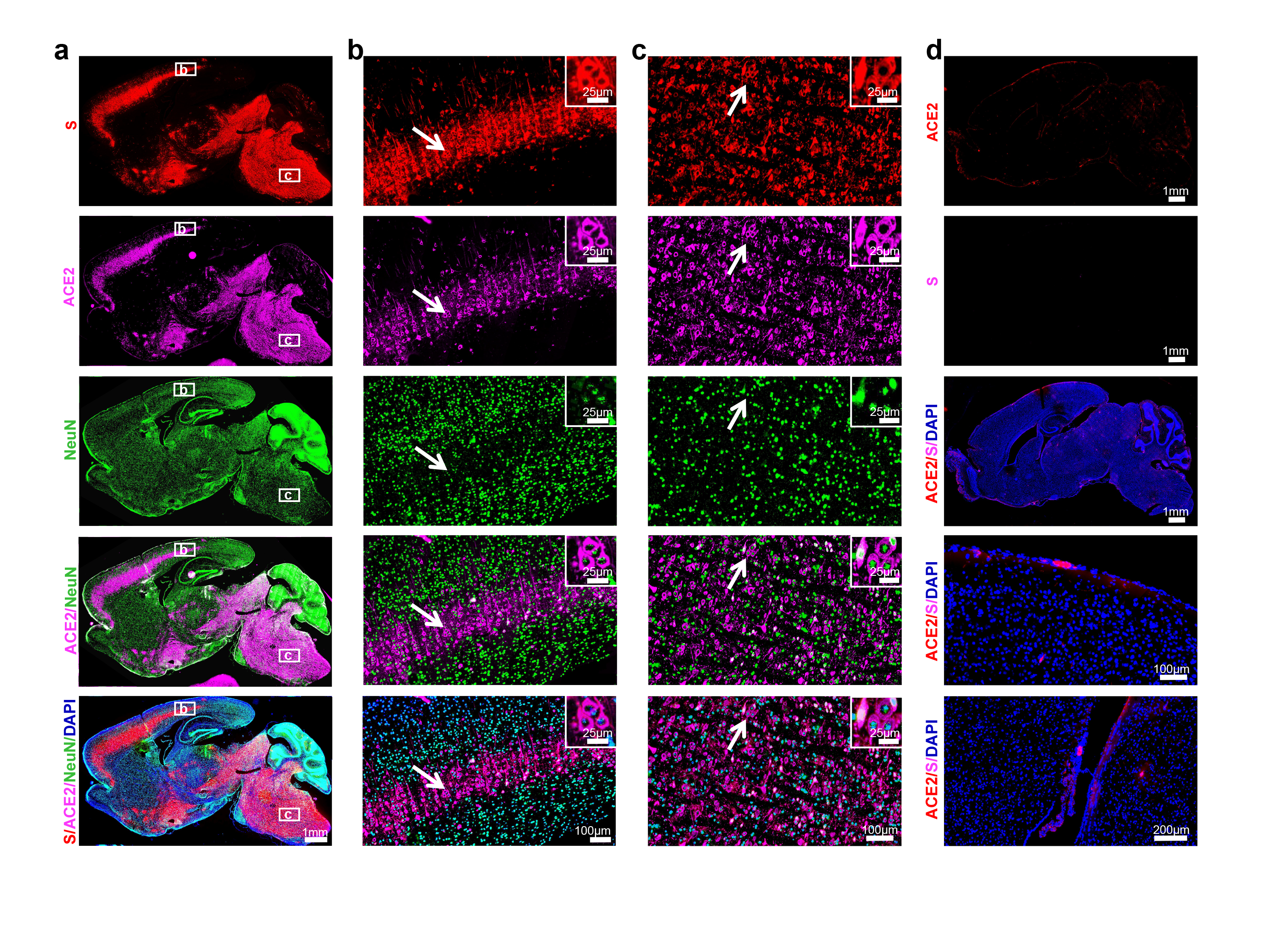
**
