# Supplementary figures and images for: SARS-CoV-2 crosses the blood–brain barrier accompanied with basement membrane disruption without tight junctions alteration
Source: Signal Transduct Target Ther. 2021 Sep 6;6:337. doi: 10.1038/s41392-021-00719-9 (PMC8419672; doi:10.1038/s41392-021-00719-9)

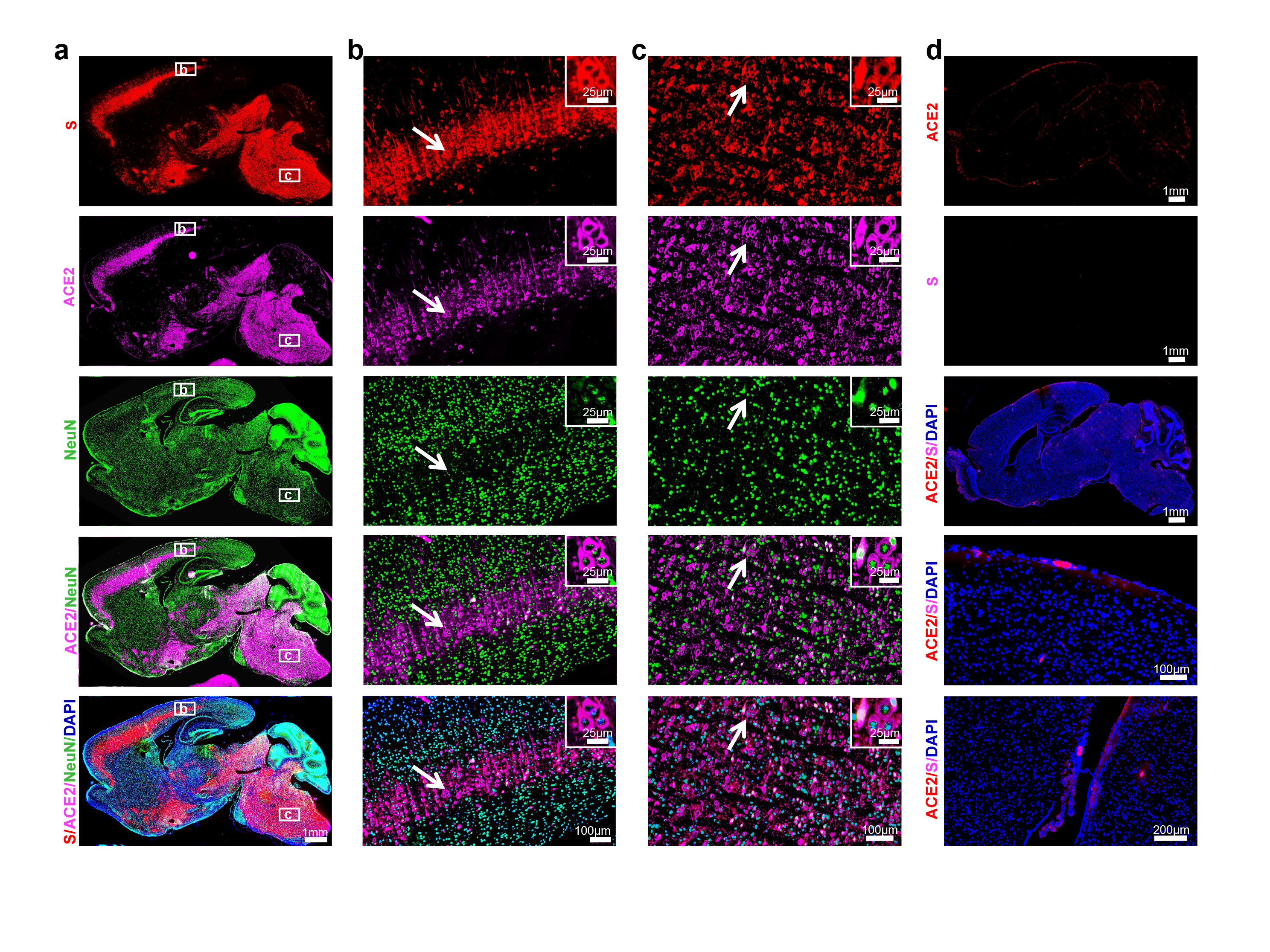

Supplement: Supplementary file 2 — Supplemental Figure 1 [file 41392_2021_719_MOESM2_ESM.tif]
